# Impact of Apricot Pulp Concentration on Cylindrical Gel 3D Printing

**DOI:** 10.3390/gels9030253

**Published:** 2023-03-21

**Authors:** Carmen Molina-Montero, Adrián Matas, Marta Igual, Javier Martínez-Monzó, Purificación García-Segovia

**Affiliations:** I-Food Group, IIAD, Universitat Politècnica de València, Camino de Vera s/n, 46022 Valencia, Spain; mamomon3@doctor.upv.es (C.M.-M.); admagi@doctor.upv.es (A.M.); marigra@upvnet.upv.es (M.I.); xmartine@tal.upv.es (J.M.-M.)

**Keywords:** printability, fruity gels, rheology, texture, bioactive compounds

## Abstract

The process of 3D food printing is a rapidly growing field that involves the use of specialized 3D printers to produce food items with complex shapes and textures. This technology allows the creation of customized, nutritionally balanced meals on demand. The objective of this study was to evaluate the effect of apricot pulp content on printability. Additionally, the degradation of bioactive compounds of gels before and after printing was evaluated to analyze the effect of the process. For this proposal, physicochemical properties, extrudability, rheology, image analysis, Texture Profile Analysis (TPA), and bioactive compounds content were evaluated. The rheological parameters lead to higher mechanical strength and, thus, a decrease in elastic behavior before and after 3D printing as the pulp content increases. An increase in strength was observed when the pulp content increased; thus, sample gels with 70% apricot pulp were more rigid and presented better buildability (were more stable in their dimensions). On the other hand, a significant (*p* < 0.05) degradation of total carotenoid content after printing was observed in all samples. From the results obtained, it can be said that the gel with 70% apricot pulp food ink was the best sample in terms of printability and stability.

## 1. Introduction

The process of 3D printing, also known as additive manufacturing (AM), allows the development of complex shapes by layer-by-layer deposition of extruded material through a nozzle [1]. Two concepts are critical in 3D food printing: (i) printability, referred to as the ease of handling and deposition of the material; (ii) buildability, the capacity to maintain its structure and dimensional stability, not only after printing, but also post-treatment process [2]. Thus, it is necessary to manipulate food inks’ rheological, structural, and mechanical properties to achieve better printability and accuracy [3]. Therefore, it is crucial to know the nature of the materials and their ingredients to be printed. In this case, fruits and vegetables are materials that cannot be extruded in their raw form due to high levels of moisture content, requiring the addition of other materials such as hydrocolloids, to increase the extrudability and viscosity of the paste to be printed [4,5,6,7]. These polymers are used to thicken or gel aqueous solutions and could improve flow properties suitable for printing with just a small amount [8]. Hydrocolloids, such as xanthan gum, kappa carrageenan, locust bean gum, and guar gum have been utilized by authors such as Pant et al. [9] and Liu et al. [10] to modify the texture of vegetable purees (garden pea, carrot, bok choy, and potato) for the purpose of 3D printing. Chen et al. [11] used inks for 3D printing of soy protein isolate with sodium alginate and gelatin. Gelatin could be a hydrocolloid of interest in preparing fruit hydrogels. Gelatin is a water-soluble, natural polymer with a high molecular weight derived from collagen. Thanks to its gelling properties, this hydrocolloid forms a microstructural network like that of polysaccharides. It also has a glossy, transparent appearance and a clean flavor profile that melts in the mouth [12,13,14].

Some authors have developed fruit-based printing ink formulations, such as Severini et al. [15] who developed inks made from mixtures of pear, kiwi, avocado, broccoli, and carrot with the addition of fish collagen as thickening agent. L. Feng et al. [16] evaluated the rheological properties of a mixture of carrot pulp, potato starch, xanthan gum, and water for printing gels with cylindrical shape. Finally, Yang et al. [17] prepared a mixture of mango juice concentrate and potato starch to evaluate the post-treatment of 3D printed gel. All of these studies incorporated a thickening agent into the formulation to carry out 3D printing.

The main advantage of this technology is its potential to customize nutritionally, sensory, and aesthetically according to consumer requirements and trends [5]. Currently, consumers demand healthier diets with more functional and bioactive ingredients [18]. Fruits and vegetables are foods with a high content of vitamins, minerals, antioxidants, sugar, and bioactive compounds and help us maintain a high quality of life [19,20]. In this scenario, a novel technology in the food sector, such as 3D printing, could play an essential role in developing formulations containing fruits and vegetables, increasing their consumption, and promoting healthy habits.

Apricot (*Prunus armeniaca* L.) is a fruit that provides an optimal combination of minerals (magnesium, calcium, iron, zinc, and copper), fibers, sugars, vitamins, and bioactive phytochemicals such as antioxidants, carotenoids, and phenols, which are of great biological importance [21,22]. Likewise, its polyphenolic compounds reduce chronic diseases and oxidative stress. All of these qualities make it a fruit of high demand from consumers; therefore, it is very interesting for developing new healthy formulations for 3D printing [23].

The purpose of this work was to study the impact of different concentrations of apricot pulp on the printability of gelatin gels. To achieve this goal, evaluation of physicochemical properties, extrudability, rheology before and after printing, image analysis, and textural properties was carried out. Moreover, the degradation of bioactive compounds of gels before and after printing was measured to analyze the effect of printing. This study enables the development of functional inks with fruits for application in food 3D printing technology.

## 2. Results and Discussion

### 2.1. Physicochemical and Rheological Analysis of Gels

Table 1 shows the values of °Brix, pH, aw, and viscoelastic parameters of the oscillatory test. Apricot pulp (AP) showed values of °Brix, pH, and aw of 10.68 ± 0.09, 3.60 ± 0.02, and 0.9805 ± 0.0007, respectively. The greater the concentration of apricot pulp in the gels, the higher the soluble solids content and the lower the pH values. Thus, °Brix and pH values of G70 gels are more similar to those of apricot pulp due to the higher concentration of apricot pulp in these samples. The water activity of the three samples showed high values very close to 1, with G70 with the lowest water activity due to a higher content of soluble solids in its composition. Sugar in gels reduces the aw, showing significant differences (*p* < 0.05) between samples.

Table 1 also shows the viscoelastic parameters of the oscillatory test of three gel samples with different concentrations of apricot pulp (30, 50, and 70%). An increase in viscoelastic parameters (G*, G′, G″, Tan δ, and η*) was observed with increasing apricot pulp in the gels (G30, G50, and G70). Values of G′ (181, 186, and 253 Pa, respectively) higher than the values of G″ (20.6, 34.2, and 57 Pa, respectively) and values of Tan δ (0.114, 0.183, and 0.226, respectively) were obtained.

The storage modulus (G′) is defined as the elastic behavior of solids, reflecting the mechanical resistance to deformation of the materials, and the loss modulus (G″) is the viscous response of the material. The loss tangent (Tan δ = G″/G′) explains the viscoelastic behavior. If Tan δ < 1, there is a predominance of the elastic behavior, and if Tan δ > 1, the viscous behavior predominates [2,24].

**Figure 1:**
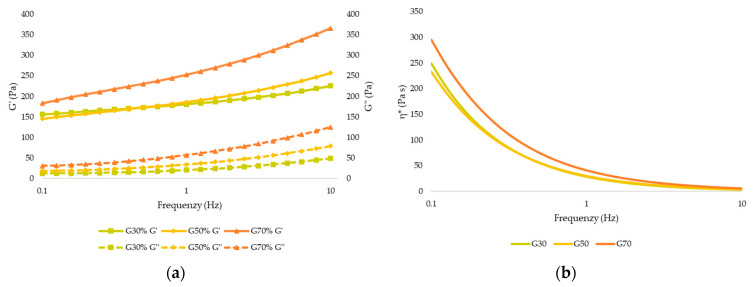
(**a**) Elastic modulus (G′) and viscous modulus (G″). (**b**) Complex viscosity (η*) of the apricot gel before 3D printing (G30: Gel 30% pulp; G50: Gel 50% pulp; G70: Gel 70% pulp).

The rheological characterization of the gel provides us information about the characteristics and behavior of the gel, as well as some sensory and textural properties [25]. Figure 1 shows the viscoelastic modulus (G′ and G″) and complex viscosity (η*) curves of the gels enriched with different concentrations of apricot pulp before 3D printing. Considering the viscoelastic properties of the gel (Figure 1a), the storage modulus G′ of the apricot pulp printing inks was higher than the loss modulus G″ throughout the studied frequency range for all samples, and the loss tangent (Tan δ) values for all apricot gel samples were less than 1, suggesting that all the gels with apricot pulp showed dominant elastic behavior [26]. The G′ and G″ values of the gels increased as a higher concentration of apricot pulp was added, indicating that the addition of apricot pulp modified the viscoelastic behavior of the gels. This increase could be associated with the ability of the sugar to attract and bind water molecules, increasing the firmness of the gel [26], with G70 being the sample most resistant to deformation. Similar results have been observed by other authors working with blueberry gelatin gels and mango jam, in which an increase in G′ and G″ moduli were observed with respect to the control [27,28]. As shown in Figure 1b, all samples presented a decrease in η* with increasing frequency being a pseudoplastic fluid. No significant difference (*p* > 0.05) between samples G30 and G50 appeared. This behavior of the samples is appropriate for 3D printing by extrusion, as the material will be able to flow easily through the nozzle due to the shear thinning that occurs when the applied stress exceeds the elastic limit and higher shape retention after 3D printing [7,29,30]. In addition, viscosity increases as the concentration of pulp in the samples increases, suggesting that the pulp content determines the viscous properties in the samples.

### 2.2. Extrudability

**Figure 2:**
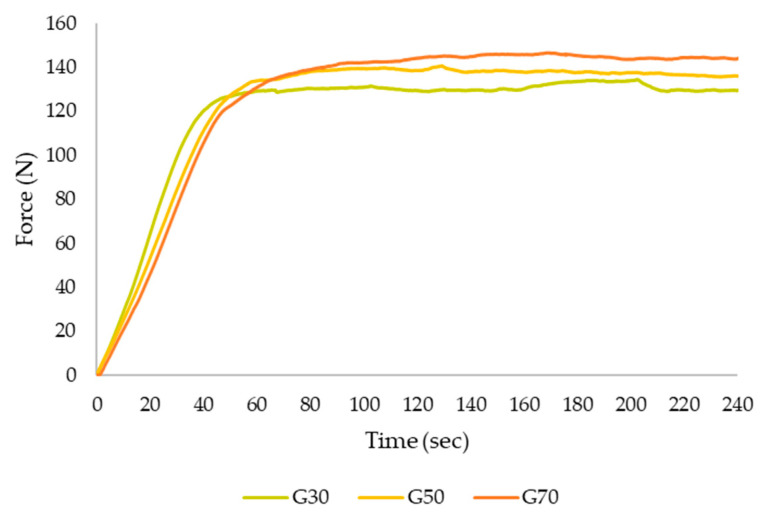
Example of force vs. time curves during forward extrusion measurement for apricot gel before 3D printing. (G30: Gel 30% pulp; G50: Gel 50% pulp; G70: Gel 70% pulp).

The extrusion test measures the compressive force required to flow through a nozzle [31]. Figure 2 shows the typical force versus time curves obtained in the extrusion test of the three apricot gels. The curves show the different periods that occur in the gel through the extrusion process. First, when the piston was pushed on the sample, the force increased steeply until reaching a maximum force which is the beginning of the extrusion through the nozzle. When the maximum force was reached, a plateau was observed, this being the average force necessary to continue the extrusion process [32].

Table 2 shows the gradient (Gr), maximum force (FM), and mean force (Fm) of the three samples after the extrusion test. The extrusion FM ranged between 130 and 144 N, the Fm between 123 and 137 N, and the gradient between 2.544 and 1.518 N/s. Significant differences (*p* < 0.05) were observed in the FM of the three samples, with G70 gels requiring the highest FM to be extruded. Therefore, an increase in the amount of apricot pulp in the gels causes a higher FM. Regarding Fm, no significant differences (*p* > 0.05) were found between samples G50 and G70, presenting very similar Fm values of 135 and 137 N, respectively. The gradient values decreased significantly (*p* < 0.05) with increasing pulp concentration in the samples. The gradient is related to the capacity of the gel to flow, where a higher gradient is related with the ability to flow more easily (less viscous). In this case, a lower percentage of apricot in formulation implies a higher gradient and higher capacity to flow through the nozzle. The gradient and FM are related to the stiffness and firmness of the gels [33].

### 2.3. Relationships of the Parameters Studied before Printing

A Pearson correlation analysis was conducted to explore the relationship between the physicochemical, rheological, and extrusion parameters of the gels (Table 3).

°Brix was significant (*p* < 0.05) and correlated negatively with aw and pH. However, it showed a positively significant (*p* < 0.05) correlation with all rheological and extrusion parameters, except for gradient. When pulp was increasingly added to the formulations, there was an increase in sugars and fiber. This sugar has hydroxyl groups that stabilize the structure of the bonding zones and promote hydrogen bonds, leaving less water available [14,34]. Nevertheless, this increased formation of hydrogen bonds led to greater firmness in the structure of the gels, which was reflected in the increase in elastic-like behavior, FM, and Fm [27].

In contrast, aw and pH showed a negatively significant correlation (*p* < 0.05) with all rheological and extrusion parameters except for the gradient. A decrease in pH is associated with a higher gel firmness and greater resistance to deformation. This effect can be explained because gelatin has an isoelectric point between 5.0 and 9.0; when the pH is far from the isoelectric point, the gel structure is stronger and more stable, having a high modulus and fracture stress [35,36,37]. On the other hand, G*, G′, G″, Tan δ, and η* show a positively significant (*p* < 0.05) correlation with each other and with FM and Fm. The gradient was significant (*p* < 0.05) and negatively correlated with rheological and extrusion parameters. Finally, FM was positively correlated with Fm.

### 2.4. Post-3D Printing

#### 2.4.1. Image Analysis

Printability is crucial in 3D food printing; it refers to the ability of a printer to print the material and preserve the object’s structure and shape [6]. Image analysis was conducted to assess the accuracy of the printer’s shape reproduction and the gel’s capability to create self-supporting layers. The parameters evaluated for a cylindrical shape after printing were its height and area.

**Figure 3:**
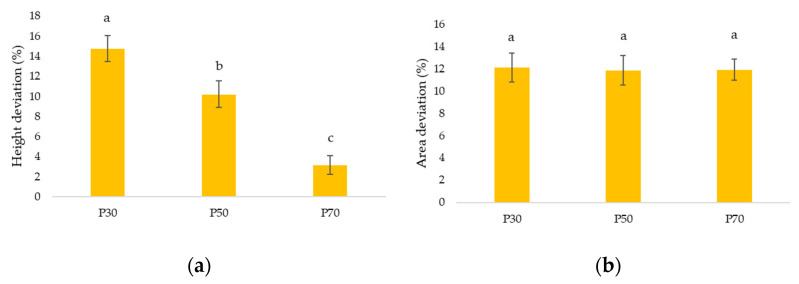
(**a**) Deviations of the height parameter of the samples. (**b**) Deviations of the area parameter of the sample (P30: Printed sample 30% pulp; P50: Printed sample 50% pulp; 70: Printed sample 70% pulp). Letters (a–c) indicate homogeneous groups according to ANOVA (*p* < 0.05).

Figure 3a shows the percent deviations (%) of the samples in height. Significant differences (*p* < 0.05) were observed among the three samples, as the percentage of apricot pulp in the samples decreased, the greater the positive deformation in height. As a result, sample P70 had less deviation in height, which corresponds to its higher rheological and extrusion values. An increased concentration of apricot pulp led to higher sugar and fiber content, resulting in better shape stability. Figure 3b shows the area deviations in percentage (%). There were no significant differences (*p* > 0.05) found between the samples, all with a positive deviation of about 12% compared to the designed cylinder.

**Figure 4:**
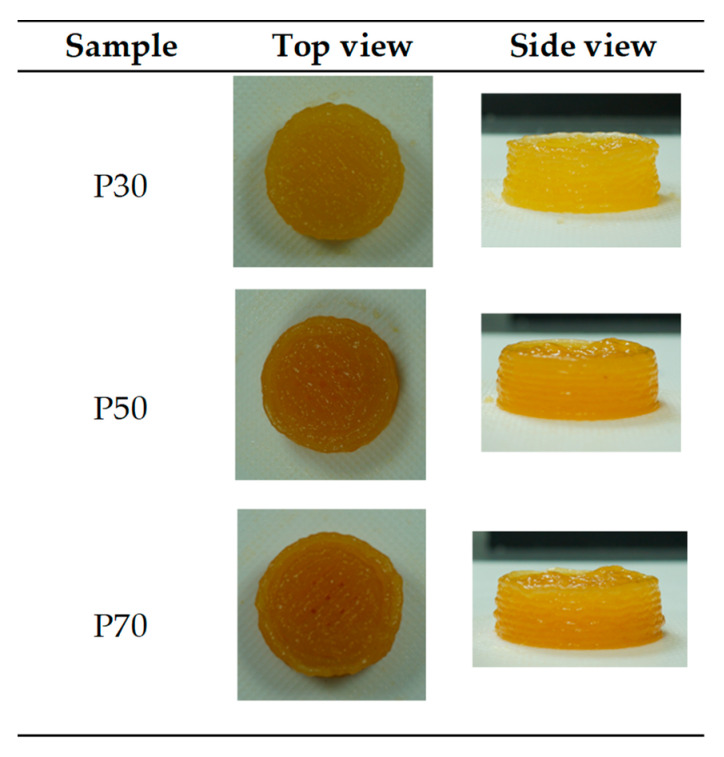
3D printed samples top and frontal view just after printing. (P30: Printed sample 30% pulp; P50: Printed sample 50% pulp; P70: Printed sample 70% pulp).

Figure 4 shows the top and front views of the printed gels with apricot pulp concentrations of 30%, 50%, and 70%. The sample P70 exhibited more defined printing lines, likely due to improved bonding in the gel resulting from higher sugar and fiber content, confirming its better stability as reported in [25]. In et al. [26] observed that gelatin gels with elevated sugar content have increased structural stability and improved printing accuracy.

#### 2.4.2. Color

Table 4 shows the apricot gel samples’ mean values and standard deviation of color coordinates of apricot gel samples. The results indicated a higher brightness in the formulations with a lower amount of apricot pulp, with no significant differences (*p* > 0.05) between samples P30 and P50. The values of a* and b* are positive, with the gels showing reddish (a*) and yellowish (b*) colorations, and phenolic compounds and carotenoids, mainly β-carotene, responsible for this coloration [38,39]. Therefore, an increase in apricot pulp concentration causes higher values of a* and b*, creating significant differences (*p* < 0.05) between samples. These results agree with Casas-Forero [40], where increases in parameters a* and b* were observed with the increasing concentration of blueberry juice cryoconcentrate in gelatin gels. Likewise, it was observed that the color intensity (C*) is dependent on the concentration of apricot pulp, P70, the sample with the highest color intensity.

The color differences between samples P30 and P50 were 8.7 ± 1.7, and those of P50 and P70 were 7.1 ± 2.4, with no significant differences (*p* > 0.05) between them. On the other hand, significant differences (*p* < 0.05) were obtained between P30 and P70, obtaining a value of 14.7 ± 2.2. As expected, the color differences were greater than 3 units (ΔE > 3) in all cases being perceptible by the human eye [41].

#### 2.4.3. Rheology after Printing

Rheological properties are usually studied to ensure successful printing, i.e., the food ink flows through the nozzle and maintains its shape and internal structure once extruded [2]. However, to improve the applicability of this technology, an additional study of the rheology after the printing process is needed to know the material’s behavior throughout the printing stages [42]. 

**Figure 5:**
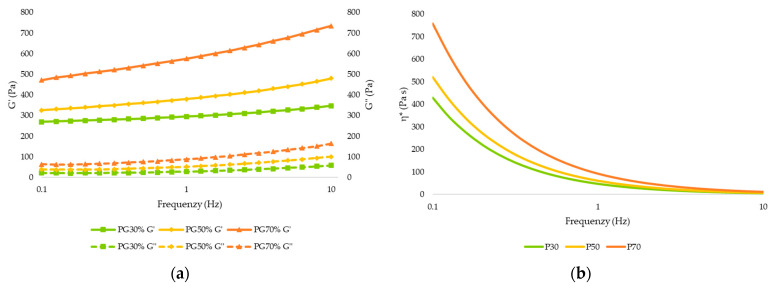
(**a**) Elastic modulus (G′) and viscous modulus (G″). (**b**) Complex viscosity (η*) of the apricot gel after 3D printing (P30: Printed sample 30% pulp; P50: Printed sample 50% pulp; P70: Printed sample 70% pulp).

Table 5 shows the rheological parameters of the gels after printing. Figure 5a shows the storage modulus (G′) and loss modulus (G″). G′ was consistently higher than the G″ as the frequency range (0.1–10 Hz) increased, with a predominance of elastic-type behavior (tan δ < 1) in the samples. In addition, it was observed that G′ and G″ increased significantly (*p* < 0.05) with increasing apricot pulp content, with P70 being more resistant to deformation. This effect could be due to a high facility for forming hydrogen bonds in the gelatin network structure due to higher sugar content, forming a stronger structure [27].

As shown in Figure 5b, the apparent viscosity of the gel after 3D printing decreases as the frequency range (Hz) increases, indicating that the gels are pseudoplastic fluids. The increase in apricot pulp content in the formulations has led to an overall increase in viscosity, which is beneficial for maintaining the printed shape.

After printing, it was observed that all rheological parameters (G*, G′, G″, and η*) increased, except Tan δ, which presented lower values as the pulp content in the samples increases. Therefore, after printing, they presented stronger elastic properties and a more solid behavior, with the difference in values between G′ and G″ being greater, increasing 100 units for each increase in concentration in the samples [34]. This increase in rheological parameters after printing may be due to the pressure exerted by the plunger when printing the sample. It can be presumed that by increasing the pressure, a more stable gel network is formed, due to a greater interconnectivity of the triple helix bonding zones per molecular chain and the stability of intermolecular hydrogen bonds is promoted [43]. Kulisie-wicz and Delgado [44] observed that, in bovine gelatin gels, when high pressures were applied, the value of G′ increased compared to gels measured at ambient pressure. This agrees with the research of Montero et al. [45] who observed an increase in G′ and G″ values when high pressures were applied to cured gelatin of cod and megrim skin compared to ambient pressure gels.

#### 2.4.4. Textural Characterization

The texture of food is a physical attribute that affects its sensory properties and structure [6]. Thus, a TPA test was conducted to assess the texture of apricot gel samples.

Table 6 shows the different TPA test parameters of the apricot gel samples. Increasing the proportion of apricot pulp in the gels intensified hardness, which improved the structure of the printed sample. Of the samples, P70 was the most resistant to deformation, followed by P50. Furthermore, sample P70 exhibited higher adhesiveness, with no significant differences (*p* > 0.05) between the other two samples. As for cohesiveness, there were no significant differences (*p* > 0.05) among the samples.

The parameter that measures a material’s ability to return to its original shape following a deformation is referred to as springiness [46]. Sample P50 presented a higher degree of springiness compared to the other samples, with significant differences (*p* < 0.05). The gumminess of the samples increased with an increase in pulp content, with a maximum value of 1.22 N recorded for P70. Conversely, the resilience of the samples decreased from 0.46 to 0.37 N.

#### 2.4.5. Relationships of the Parameters Studied after Printing

Pearson correlations were observed between rheological parameters, height and area deviations, and textural parameters (Table 7).

All rheological parameters showed a positively significant (*p* < 0.05) correlation with each other and a negatively significant (*p* < 0.05) correlation with the height deviation. As the elastic behavior of the specimen increases, the height deviation is lower. According to In et al. [26], high values of G′ and Tan δ maintain denser structures with high dimensional stability after printing. G* shows a positively significant (*p* < 0.05) correlation with hardness and gummies but a negatively significant (*p* < 0.05) correlation with adhesiveness, cohesiveness, and resilience. In addition, height deviation shows a negatively significant correlation (*p* < 0.05) with hardness. Consequently, a higher hardness in the samples will produce a higher resistance to deformation and, therefore, a lower height deviation after printing. There were no significant correlations (*p* > 0.05) for the parameters of springiness and area deviation, but there were significant correlations for hardness, which presented a positively significant correlation with gumminess (*p* < 0.05).

A Pearson correlation analysis was performed to study the parameters before and after printing. °Brix showed a significant positive correlation (*p* < 0.05) with hardness (0.9936) and gumminess (0.9640) and with all rheological parameters (0.9430; *p* < 0.05) after 3D printing but negatively with the rest of the TPA parameters (−0.8287; *p* < 0.05) and height deviation (−0.9893). Moreover, FM (0.9486) and Fm (0.8880) presented a positively significant correlation (*p* < 0.05) with hardness. This high consistency could be related to the addition of pulp since it leads to higher sugar content and a greater stabilization of the collagen, generating greater connections and reinforcing the gelatin network [27]. Consequently, a gel with a more elastic behavior is obtained and therefore, there is a smaller deviation in height and a greater replication of the printed figure.

### 2.5. Effect of 3D Printing on Bioactive Compounds in Apricot Gels

Nutritionally, apricot fruit contains many phytochemicals, such as carotenoids, flavonoids, lycopene, and other antioxidant compounds, which are essential for their biological value [22,47]. Carotenoids are compounds with low water solubility and are chemically unstable. Their oxidative degradation is triggered by light, temperature, and/or pH extremes in the presence of oxygen, compromising their benefits and limiting their use in the food industry [18,48,49].

Table 8 shows the apricot gels’ bioactive compounds studied (total carotenoids, lycopene, and total phenols) before and after 3D printing. The values of total carotenoids, lycopene, and total phenols of apricot pulp were 2.92 (0.03) mgβ-carotene/100 g, 0.93 (0.04) mg/100 g, and 81.9 (1.4) mgGA/100 g, respectively, being in the same range of values as Fatima et al. [50]. A 20% increase of apricot pulp in the formulations increased the total values of carotenoids, lycopene, and total phenols, but after printing there was a significant decrease (*p* < 0.05) in the total carotenoid content, this decrease being higher in P50 at 19.34%, than for P30 and P70, which showed a decrease of 16%. This decrease may be due to a destruction of the cellular structure after 3D printing, increasing the oxidative degradation and, therefore, the porosity of the printed material [51]. Finally, no change in lycopene and total phenol content was observed after printing.

## 3. Conclusions

This study investigated the effects of an apricot pulp addition to gel printability and physiochemical properties before and after 3D printing. The addition of different percentages of apricot pulp in the samples changed the physicochemical (°Brix, pH, aw, color), rheological, extrudable, and textural properties. When the pulp concentration was increased, the pH decreased, while the °Brix and elastic modulus increased. The sample with the highest resistance to deformation was G70, while G30 had a greater ability to flow through the nozzle due to a higher gradient. After printing, stronger elastic properties and a more solid behavior of the gels were observed, due to the pressure increases produced during printing. This resulted in gels with a higher number of triple helix bonding zones per molecular chain that were structurally more stable. This behavior is ideal for constructing the printed figures and preserving structural integrity, with P70 as the sample that showed the lowest percentage of height deviation and the best printing definition, as well as higher hardness and gumminess. In addition, P70 was the sample with the highest content of bioactive compounds.

## 4. Materials and Methods

### 4.1. Raw Materials

Apricot pulp and chemical composition (Table 9) was sourced from Jumel Alimentaria S.A. (L’Alqueria de la Comtessa, Spain), and bovine gelatin was provided by Sosa Ingredients S.L. (Barcelona, Spain).

### 4.2. Apricot Gel Preparation

The gels were made using 5% bovine gelatin (220 BLOOM) with varying amounts of apricot pulp: 30% (G30), 50% (G50), and 70% (G70). The bovine gelatin was dissolved in mineral water heated to 60 °C, then cooled to 35 °C. The apricot pulp was then mixed in and stirred until evenly distributed. The mixture, totaling 40 mL, was filled into 100 mL syringes and refrigerated for 40 min at 4 °C. Afterward, they were brought to room temperature (25 °C).

### 4.3. The 3D Printing Process

The gels (G30, G50, and G70) were printed using a commercial 3D printer (BCN 3D+ from BCN3D Technologies, Barcelona, Spain) with a pasta extruder nozzle specifically designed for food materials. The printing system consisted of an extrusion system and an X-Y-Z positioning system controlled by stepper motors. Printing was done at 25 °C. A 3 cm diameter and 1 cm height cylinder was designed in Tinkercad (free software from Autodesk, Inc., San Rafael, CA, USA), and the printing parameters were set using the Slic3r program (free software developed by Alessandro Ranellucci): speed 20 mm/s, layer height 1.63 mm, and 100% rectilinear infill. A 1.63 mm diameter nozzle was used for all samples. The printed samples were labeled as P30, P50, and P70 to indicate the apricot pulp content of 30%, 50%, and 70%, respectively.

### 4.4. Analysis

#### 4.4.1. Rheological Properties

Oscillatory test of gels before and after printing were performed using a Kinezus Pro + rotational rheometer (Malvern Instruments, Worcestershire, UK) equipped with a 40 mm diameter parallel-plate geometry with a 1.0 mm gap between plates. rSpace software (Malvern Instruments, Worcestershire, UK) was used for data acquisition and evaluation. A sample was loaded in the geometry and rested to equilibrate for 5 min to achieve a temperature equilibrium (25 °C). The linear viscoelastic region for all the samples was determined using an amplitude sweep test. The initial shear stress ranged from 0.1% to 100% at the end at 1 Hz frequency. An oscillatory test was carried out at a fixed strain of 1 Pa and a frequency range of 0.1 to 10 Hz. From this frequency, sweep was obtained for the elastic modulus (G′ (Pa)) related to the material response as a solid; viscous modulus (G″ (Pa)) related to the material response as a fluid; complex modulus (G* (Pa)) related to the total resistance of the material against applied deformation; the loss tangent, or Tan δ defined as the ratio of G″ to G′ to different frequency values (Hz); and complex viscosity (η* (Pa∙s)) defined as the total resistance of a material to dynamic shearing. All tests were performed in triplicate.

#### 4.4.2. Extrusion Test

The printing conditions (velocity, temperature, syringe, and nozzle diameter) were replicated in the extrusion test. An extrusion test was performed using a TA.XT.plus tex-turometer (Stable Micro Systems, Godalming, Surrey, UK) and Texture Exponent 32 software (Stable Micro Systems, Godalming, Surrey, UK). The printer plunger, syringe with sample, and nozzle were placed on the texturometer and a cylindrical press attachment were used to stabilize the syringe. The test conditions were 0.04 mm/s downstroke speed, and 10 mm distance travelled [52]. From resulting curves, Force/time, Gradient (Gr (N/s)), Maximum Force (FM (N)), and Mean Force (Fm (N)) parameters were obtained. Gr is the force per second applied by the plunger until the maximum force is reached, FM is the force at which the sample begins to extrude through the nozzle, and Fm is the force required to continue the extrusion.

#### 4.4.3. Physicochemical Analysis

Additionally, °Brix and pH were evaluated for apricot pulp and gels, but water activity (aw) was evaluated only for gels. The soluble solids content, expressed as °Brix, was measured with a PAL-1 digital refractometer (ATAGO Co., Ltd., Tokyo, Japan). The pH values were measured with a HI99163 pH meter (Hanna Instruments Inc., Woonsocket, RI, USA). The sample water activity (aw) was analyzed by AquaLab 4TE LabFerrer equipment (Decagon Devices, Inc., Pullman, WA, USA).

#### 4.4.4. Color

The color of the apricot gel samples after printing was determined by the CIEL*a*b* color space method. Color coordinates were obtained using a Konica Minolta CM-700d colorimeter (Konica Minolta CM-700d/600d series, Tokyo, Japan) with standard illuminant D65 and a visual angle of 10°. Results were obtained in terms of L* (brightness: L* = 0 (black), L* = 100 (white)), a* (−a* = green, +a* = red), and b* (−b* = blue, +b* = yellow), ac-cording to the CIEL*a*b* system [53]. From color coordinates, Chroma (C*_ab_, saturation) and hue angle (h*_ab_) were calculated. Additionally, the differences in color between the samples were evaluated.

#### 4.4.5. Image Analysis

**Figure 6:**
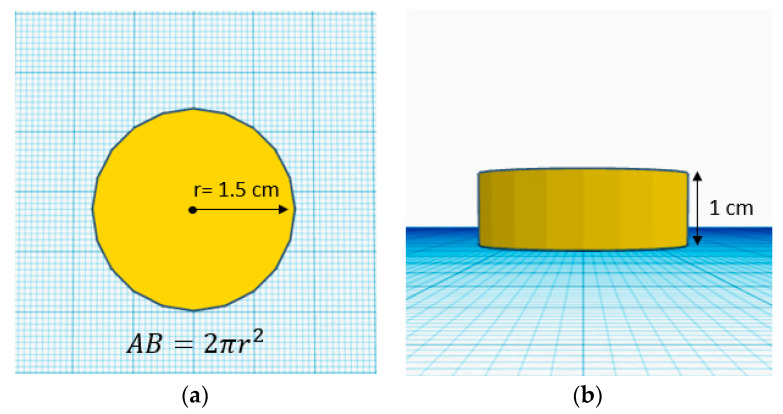
(**a**) Cylinder base area; (**b**) Cylinder height.

Photographs were taken of the top and front of each cylinder of printed apricot gel. These images were analyzed using ImageJ software (ImageJ, NIH, Washington, DC, USA) to evaluate the dimensions of the figures. The base area was measured in the top view images and the height was measured in the front view images, as illustrated in Figure 6. The differences between the printed figures and the target figures were calculated as a metric of dimension variation. The percentage of variation of the samples was calculated by the difference of the characteristic dimensions (area and height) printed figures (*DPF*) and the dimensions of target figure (*DTF*) divided by the target dimensions multiplied by 100 (Equation (1)).

(1) $$\%\;Variation\;of\;dimensions=\frac{\left(DPF-DTF\right)}{DTF}\times 100$$

#### 4.4.6. Textural Characterization

The texture profile analysis (TPA) of apricot pulp cylinders after 3D printing was performed using a TA.XT.plus texturometer (from Stable Micro Systems, Godalming, Surrey, UK). The TPA was conducted using a 4 cm diameter cylindrical aluminum probe and a 50 kg load cell. The analysis was performed with double compression at a speed of 0.5 mm/s with a 5 s rest period between cycles and a 40% deformation of the original length. The parameters of hardness, adhesiveness, cohesiveness, springiness, gumminess, and resilience were extracted using the Texture Exponent 32 program (from Stable Micro Systems, Godalming, Surrey, UK). All samples were tested in triplicate.

#### 4.4.7. Bioactive Compounds Determination

Total carotenoids, lycopene, and total phenols of apricot gels’ before and after 3D printing were determined. Total carotenoids (TC) were extracted with a solvent hexane/acetone/ethanol mixture following the Olives-Barba et al. method [54]. The spectrophotometric reference method of AOAC [55] was used for quantification. The absorbance was measured at 446 nm in a UV-3100PC spectrophotometer (VWR, Leuven, Belgium). The TC content was expressed as mg of β-carotene (Sigma-Aldrich, Steinheim, Germany) per 100 g of sample (mgβ-carotene/100 g). From the TC extract, lycopene (LP) was determined. For it, sample absorbance was measured at 503 nm. LP content was expressed as mg/100 g of sample, calculated according to Khamis et al. [56].

For total phenols (TP) analysis, methanol was used for the sample extraction, and the Folin-Ciocalteu method was performed according to the method described by Igual et al. [57]. Briefly, 1 g of sample was mixed with 5 mL methanol, 0.5 mL HCl 5 N, and NaF 2 mM and centrifugated at 10,000 rpm, 4 °C, for 10 min using an Eppendorf centrifuge (Eppendorf, Hamburg, Germany). From the supernatant, 250 µL was mixed in a 10 mL volumetric flask with 1.25 mL Folin-Ciocalteu reagent and stored in a dark place for 8 min. Afterwards, 3.75 mL Na_2_CO_3_ with a concentration of 7.5% was added and further stored for 120 min. The samples’ absorbance was read with a UV-visible spectrophotometer (UV-3100PC, VWR, Leuven, Belgium) at 765 nm and expressed as mg gallic acid/100 g sample (mgGA/100 g).

### 4.5. Statistical Analysis

An analysis of variance (ANOVA), with a confidence 95% level (*p* < 0.05), by the Statgraphics Centurion XVIII Software, version 18.1.13, was applied to evaluate the differences between apricot gels. The method used to discriminate between means was Fisher’s least significant difference (LSD) procedure. Correlations between parameters before and after 3D printing, with a significance level of 95%, were studied using Pearson’s coefficients.

## Figures and Tables

**Table 1:** Mean values (and standard deviations) of °Brix, pH, a_w_, and rheological parameters (G*, complex modulus; G′, storage modulus; G″, loss modulus; Tan δ, loss tangent; and η*, apparent viscosity) of apricot gels before 3D printing.

	G30	G50	G70
°Brix	8.7 (0.2) ^c^	11.4 (0.3) ^b^	14.5 (0.3) ^a^
pH	4.457 (0.006) ^a^	4.21 (0.02) ^b^	4.013 (0.006) ^c^
a_w_	0.9964 (0.0006) ^a^	0.9946 (0.0003) ^b^	0.9925 (0.0003) ^c^
G* (Pa)	182 (4) ^b^	190 (7) ^b^	259 (20) ^a^
G′ (Pa)	181 (4) ^b^	186 (7) ^b^	253 (20) ^a^
G″ (Pa)	20.6 (0.6) ^c^	34.2 (0.4) ^b^	57 (4) ^a^
Tan δ	0.114 (0.006) ^c^	0.183 (0.005) ^b^	0.226 (0.006) ^a^
η* (Pa∙s)	28.9 (0.5) ^b^	30.2 (1.2) ^b^	41 (3) ^a^

The letters (a–c) in rows indicate the homogeneous groups according to ANOVA (*p* < 0.05). (G30, Gel 30% pulp; G50, Gel 50% pulp; G70, Gel 70% pulp).

**Table 2:** Mean values (and standard deviations) of extrusion parameters (Gr, Gradient; FM, maximum force; Fm, mean force) of apricot gel before 3D printing.

	G30	G50	G70
Gr (N/s)	2.5 (0.3) ^a^	2.12 (0.06) ^ba^	1.5 (0.5) ^b^
F_M_ (N)	130 (2) ^c^	139 (2) ^b^	144 (3) ^a^
F_m_ (N)	123 (3) ^b^	135 (3) ^a^	137 (2) ^a^

The letters (a–c) in rows indicate the homogeneous groups according to ANOVA (*p* < 0.05). (G30, Gel 30% pulp; G50, Gel 50% pulp; G70, Gel 70% pulp).

**Table 3:** Pearson correlation coefficients among °Brix, aw, pH, rheological parameters (G*, G′, G″, Tan δ, and η*), and extrusion parameters (G, F_M_, and F_m_) of gels.

	a_w_	pH	G*	G′	G″	Tan δ	η*	G_r_	F_M_	F_m_
°Brix	−0.9791 *	−0.9926 *	0.8945 *	0.8845 *	0.9883 *	0.9766 *	0.8945 *	−0.8561 *	0.9430 *	0.8670 *
a_w_		0.9801 *	−0.8754 *	−0.8653 *	−0.9746 *	−0.9677 *	−0.8754 *	0.8069 *	−0.9033 *	−0.8369 *
pH			−0.8451 *	−0.8333 *	−0.9715 *	−0.9939 *	−0.8452 *	0.8449 *	−0.9525 *	−0.9047 *
G*				0.9997 *	0.9448 *	0.7934 *	1.0000 *	−0.8010 *	0.7287 *	0.6262 *
G′					0.9373 *	0.7798 *	0.9997 *	−0.7928 *	0.7147 *	0.6116 *
G″						0.9488 *	0.9448 *	−0.8692 *	0.8897 *	0.8150 *
Tan δ							0.7935 *	−0.8419 *	0.9548 *	0.9211 *
η*								−0.8010 *	0.7288 *	0.6263 *
G_r_									−0.8224 *	−0.7734 *
F_M_										0.9221 *

* Correlation is significant at 0.05.

**Table 4:** Color parameters L*, a*, b*, C*, and h* of apricot gel samples.

	P30	P50	P70
L* (D65)	51.5 (3.2) ^a^	48.1 (2.4) ^a^	42.1 (0.6) ^b^
a* (D65)	5.2 (0.6) ^c^	8.4 (0.4) ^b^	10.7 (0.2) ^a^
b* (D65)	33.4 (1.4) ^c^	40.6 (0.9) ^b^	43.1 (1.6) ^a^
C*	33.8 (1.4) ^c^	41.4 (1.2) ^b^	44.4 (1.5) ^a^
h*	1.418 (0.015) ^a^	1.366 (0.007) ^b^	1.326 (0.012) ^c^

The letters (a–c) in rows indicate the homogeneous groups according to ANOVA (*p* < 0.05). (P30: Printed sample 30% pulp; P50: Printed sample 50% pulp; P70: Printed sample 70% pulp).

**Table 5:** Rheological parameters (G*, complex modulus; G′, storage modulus; G″, loss modulus; Tan δ, loss tangent; and η*, apparent viscosity) of apricot gel after 3D printing.

	P30	P50	P70
G* (Pa)	296 (18) ^c^	384 (34) ^b^	582 (26) ^a^
G′ (Pa)	295 (18) ^c^	380 (34) ^b^	575 (26) ^a^
G″ (Pa)	28.2 (0.4) ^c^	52 (3) ^b^	87 (2) ^a^
Tan δ (°)	0.095 (0.005) ^c^	0.137 (0.005) ^b^	0.153 (0.003) ^a^
η* (Pa s)	47 (3) ^c^	61 (5) ^b^	92 (4) ^a^

The letters (a–c) in rows indicate the homogeneous groups according to ANOVA (*p* < 0.05). (P30, Printed sample 30% pulp; P50, Printed sample 50% pulp; P70, Printed sample 70% pulp).

**Table 6:** TPA test parameters.

	P30	P50	P70
Hardness (N)	1.04 (0.09) ^c^	1.39 (0.16) ^b^	1.73 (0.15) ^a^
Adhesiveness (N·s)	−0.45 (0.07) ^a^	−0.38 (0.12) ^a^	−0.71 (0.16) ^b^
Cohesiveness	0.78 (0.02) ^a^	0.75 (0.02) ^ba^	0.74 (0.06) ^b^
Springiness	0.88 (0.02) ^b^	0.91 (0.03) ^a^	0.88 (0.02) ^b^
Gummies (N)	0.83 (0.06) ^b^	1.06 (0.14) ^a^	1.22 (0.22) ^a^
Resilience	0.46 (0.03) ^a^	0.44 (0.02) ^a^	0.37 (0.03) ^b^

The letters (a–c) in rows indicate the homogeneous groups according to ANOVA (*p* < 0.05). (P30%, printing gel with 30% apricot pulp; P50%, printing gel with 50% apricot pulp; P70%, printing gel with 70% apricot pulp).

**Table 7:** Pearson correlation coefficients among rheological parameters (G*, G′, G″, Tan δ, and η*), height deviation (HD), area deviation (AD), and TPA parameters (hardness, adhesiveness, cohesiveness, springiness, gumminess, and resilience) of gels.

	G′	G″	Tan δ	η*	HD	AD	H	A	C	S	G	R
G*	1.0000 *	0.9889 *	0.8491 *	1.0000 *	−0.9700 *	−0.1234	0.9635 *	−0.8013 *	−0.8245 *	−0.1454	0.9283 *	−0.9019 *
G′		0.9883 *	0.8473 *	1.0000 *	−0.9692 *	−0.1236	−0.9626 *	−0.8025 *	−0.8243 *	−0.1472	0.9273 *	−0.9030 *
G″			0.9159 *	0.9888 *	−0.9897 *	−0.1244	−0.9897 *	−0.7397 *	0.8286 *	−0.0535	0.9585 *	−0.8508 *
Tan δ				0.8497 *	−0.9190 *	−0.1137	0.9532 *	−0.4718	−0.7803 *	0.2385	0.9532 *	−0.6543 *
η*					−0.9699 *	−0.1235	0.9635 *	−0.8013 *	−0.8245 *	−0.1454	0.9282 *	−0.9019 *
HD						0.1200	−0.9816 *	0.7140 *	0.7839 *	0.0237	−0.9383 *	0.8169 *
AD							−0.1213	0.0117	0.0033	−0.2775	−0.0543	0.0852
H								−0.6457 *	−0.8077 *	0.0730	0.9703	−0.8098 *
A									0.7151 *	0.5358	−0.6607 *	0.7963 *
C										0.0733	−0.9013 *	0.7362 *
S											0.1004	0.2531
G												−0.7972 *

* Correlation is significant at 0.05.

**Table 8:** Mean values (and standard deviations) of total carotenoids (TC, mgβ-carotene/100 g), lycopene (LP, mg/100 g), and total phenols (TP, mgGA/100 g) of studied samples.

Sample	TC	LP	TP
G30	0.573 (0.013) ^cA^	0.294 (0.008) ^cA^	41.6 (0.3) ^cA^
G50	0.858 (0.012) ^bA^	0.356 (0.012) ^bA^	48.9 (1.4) ^bA^
G70	1.279 (0.012) ^aA^	0.451 (0.012) ^aA^	59.2 (0.8) ^aA^
P30	0.481 (0.012) ^cB^	0.287 (0.007) ^cA^	41.1 (0.3) ^cA^
P50	0.692 (0.004) ^bB^	0.358 (0.003) ^bA^	47.6 (0.4) ^bA^
P70	1.07 (0.02) ^aB^	0.452 (0.005) ^aA^	58.1 (1.2) ^aA^

For each bioactive compound, the same superscript small letter within column indicates homogeneous groups established using ANOVA (*p* < 0.05) compared to samples in gels or printed sample. For each sample and bioactive compound, the same capital letter in superscript within column indicate homogeneous groups established using ANOVA (*p* < 0.05) compared to gels and printed sample. G30: Gel 30% pulp, G50: Gel 50% pulp, G70: Gel 70% pulp, P30: Printed sample 30% pulp, P50: Printed sample 50% pulp, and P70: Printed sample 70% pulp.

**Table 9:** Chemical composition of apricot pulp with average values per 100 g.

Chemical Composition Apricot Pulp	Average Values per 100 g
Energy value	43 Kcal/180 Kj
Fat	0.1 g
of which saturated fatty acids	<0.1 g
Carbohydrates	8.5 g
of which sugars	8 g
Dietary fiber	2.5 g
Proteins	0.8 g
Sodium	<0.1 g

## Data Availability

Not applicable.
